# Examining the impact of social media on youth vaping behavior in China: an analysis of the mediating role of perceptions of policy enforcement

**DOI:** 10.3389/fpubh.2025.1524524

**Published:** 2025-04-09

**Authors:** Tong-Chen Lucas Wang, Mei-Juan Zhang, Hualin Zhang

**Affiliations:** ^1^School of Media and Communication, Shenzhen University, Shenzhen, China; ^2^Institute of Global Communication, Shenzhen University, Shenzhen, China; ^3^Division of Arts, Shenzhen University, Shenzhen, China

**Keywords:** youth vaping, e-cigarettes control policy, social media exposure, perceived enforcement of tobacco control policy, risk and benefits perception

## Abstract

**Introduction:**

Research has established that exposure to media and the perceived enforcement of policies can influence outcomes related to (un)healthy behaviors. However, little is known about the underlying processes that may mediate the relationship. The Knowledge-Attitude-Practice (KAP) model serves as an important framework for examining health cognition and behavior change. It asserts that knowledge underpins beliefs, attitudes drive motivation, and practices reflect behaviors. In the realm of e-cigarette cessation, this study investigates the influence of media exposure on perceptions of policy enforcement, which in turn affects risk-benefit evaluations and behavioral outcomes.

**Methods:**

Data for this study were collected in 2024 from an online questionnaire survey (*N* = 724) conducted in Guangdong China, with participants aged 18 to 30. We primarily employ methods such as mediating effect testing and regression analysis to conduct our data analysis.

**Results:**

The findings suggest that social media exposure, perceived policy enforcement, and perceptions of risks and benefits collectively influence youth vaping behaviors through various mediating pathways. Specifically, the results indicate that exposure to social media has a positive effect on the perceived enforcement of tobacco control policy. This perception, in turn, positively affects both risk and benefit perceptions, thereby either decreasing the likelihood of vaping through heightened perceived risks or increasing it through enhanced perceived benefits.

**Discussion:**

The study highlights the impact of social media content concerning e-cigarettes, noting that both ambiguous advertising and health education materials can enhance the perceived enforcement of tobacco control policy. Furthermore, we investigate the impact of information shared across various social media platforms on vaping behaviors and perceptions of tobacco control policy enforcement. Implications and limitations are discussed.

## 1 Introduction

The Electronic Nicotine Delivery System (ENDS), invented in China in 2003, utilizes battery-operated devices to aerosolize liquid that contains nicotine (1, 2). Initially promoted as safer alternatives to traditional cigarettes, e-cigarettes are now subject to increasing global scrutiny due to their associated toxicity risks, particularly among young people. Research indicates that their acute toxicity may surpass that of conventional cigarettes, with nicotine exposure contributing to heightened addiction and subsequent tobacco use, thereby increasing the risks of cardiovascular diseases, chronic obstructive pulmonary disease (COPD), cancer, and premature death (3, 4).

Internationally, the implementation of smoke-free policies—including bans, health warnings, advertising restrictions, and taxation—has led to a decrease in both traditional cigarette smoking and e-cigarette usage (5, 6). Although China has recently enacted regulations concerning e-cigarettes (1), its tobacco control measures are still less rigorous compared to those in Singapore and Hong Kong, resulting in a slower decline in smoking rates. From 1990 to 2019, the reduction in China's smoking rate was notably behind the global average (7). Alarmingly, the prevalence of smoking among youth remains significant, with rates of 27.7% for males and 2.0% for females, and 56.2% of youth initiating smoking by the age of 18 (9). This situation highlights the urgent need for policies targeting youth within global public health initiatives.

This study employs the Knowledge-Attitude-Practice (KAP) model (10) to examine the interactions between young people's perceptions of tobacco policy enforcement, their exposure to social media, and their evaluations of risks and benefits. The KAP framework, widely utilized in health behavior research (11–14), elucidates the influence of social media on perceptions of policy enforcement in our research. Mediation analyses reveal indirect effects, demonstrating that perceived enforcement can affect vaping behavior by modifying risk-benefit assessments.

Existing research in health communication in China has explored various aspects, including policy implementation (15, 16), public attitudes (17, 19), and drivers of perception (20). However, a significant gap exists in the literature, as most studies have concentrated on policy design and public attitudes while largely overlooking perceptions of enforcement efficacy. This research seeks to fill this gap by investigating perceived enforcement as both an independent variable and a mediator. The findings indicate that variations in perceptions of enforcement are predictive of the likelihood of vaping, thereby contributing to the enrichment of the KAP model and bolstering advocacy for enhanced tobacco control measures.

## 2 Literature review

### 2.1 The Knowledge, Attitude, Practice model

The Knowledge, Attitude, Practice (KAP) model provides a framework for understanding the development of health-related behaviors through the processes of knowledge acquisition, attitude formation, and behavioral practices (10). This model has been extensively utilized in the context of vaping research, with various studies indicating that an increase in knowledge is associated with negative attitudes toward vaping and a greater likelihood of cessation (21–24). Furthermore, demographic variables such as gender (25, 26), older age (27, 28), lower educational attainment (29), and socioeconomic status (30) have been identified as predictors of vaping behaviors. Nevertheless, current literature has not sufficiently examined the underlying mechanisms that influence attitudes toward vaping.

A significant gap exists in understanding the inadequate awareness among vapers regarding the risks associated with e-cigarettes, which contributes to continued usage (31–34). For example, a lack of awareness about the potential harms and skepticism toward regulatory measures have impeded efforts to reduce vaping among Chinese middle school students (35). While previous research has focused on perceived risks and benefits of vaping, it has largely overlooked the perceptions surrounding policy enforcement.

This study aims to apply the KAP model to investigate youth vaping behavior, with an emphasis on the influence of social media on exposure to information about e-cigarettes (K), the perceptions of risks and benefits as well as policy enforcement (A), and the resultant behavioral practices (P). The research seeks to elucidate how social media shapes the psychological perceptions that affect vaping behaviors.

### 2.2 Perceived enforcement of tobacco control policy

Perceived Policy Enforcement (PPE) refers to individuals' assessments of the effectiveness of policies (36) and plays a significant role in shaping tobacco-related behaviors through two main aspects: the strictness of policies and perceptions of enforcement. Research has shown that PPE is essential in decreasing youth smoking rates, particularly through school policies (37, 38) and state-level initiatives (39, 40). Studies indicate that adolescents' views on the enforcement of local regulations are inversely related to smoking prevalence, with anti-smoking norms acting as a mediating factor (41). Furthermore, the media plays a crucial role in enhancing PPE through the dissemination of information, as social media transforms public discussions and perceptions of enforcement (42–45). This phenomenon aligns with the KAP model, where media influences PPE, which in turn affects youth behaviors.

While existing research primarily focuses on traditional cigarettes, there are significant gaps in understanding the implications for e-cigarettes. Three key research priorities emerge: (1) Examining indirect mechanisms: Understanding how PPE mediates vaping behaviors could enhance the KAP model and inform policy modifications. (2) Investigating risk-benefit perceptions: Misunderstandings regarding the safety of e-cigarettes (46–50) may interact with PPE, necessitating further investigation. (3) Exploring the role of social media: Given its significance as a primary source of information for youth (51–53), the impact of social media on PPE and vaping requires empirical scrutiny.

Additionally, PPE influences broader health behaviors, with stronger perceptions of enforcement linked to healthier choices, including lower smoking rates (54, 55). In the context of vaping, PPE may discourage e-cigarette use by shaping public attitudes. Within the KAP framework, PPE acts as an attitudinal factor that connects knowledge to practices. While media significantly shapes PPE (56–60), the mediating variables between PPE and behaviors remain largely underexplored. Investigating these pathways could enhance the predictive capabilities of the KAP model and guide targeted interventions. This study aims to thoroughly explore the antecedent factors (such as social media) and subsequent mediators to clarify the role of PPE in youth vaping behaviors.

### 2.3 Social media and perceived enforcement of tobacco control policy

In modern information environments, social media plays a crucial role in engaging young people with content related to policies, influencing their views on enforcement through both active participation and algorithm-driven exposure (61). For example, platforms such as Weibo contribute to increasing public awareness of environmental policies (62). This research specifically investigates the context of e-cigarette regulation in China, aiming to fill existing gaps in understanding how different types of social media platforms affect perceptions of tobacco control enforcement. Previous studies have indicated that social media enhances the understanding of norms and perceptions regarding smoking (63, 64), yet they often overlook the unique dynamics and content attributes of specific platforms that contribute to these effects. This study will explore the variations among platforms and the characteristics of content that influence perceptions of enforcement within Chinese social media.

Additionally, social media messaging has a direct effect on health behaviors (18, 65–67, 69, 70). In the context of vaping, exposure to e-cigarette advertisements has been shown to significantly increase the likelihood of usage (71–74). However, prior research has not effectively identified which types of messages across different platforms exert the strongest influence on behaviors. This study seeks to examine the connections between different Chinese social media platforms and adolescent vaping in order to identify the most impactful characteristics of both the platforms and the messages, thereby enhancing the understanding of how digital media influences policy perceptions and behavioral outcomes.

### 2.4 The role of perceived risks/benefits

Risk perception pertains to an individual's evaluation of health risks associated with specific behaviors, whereas benefit perception relates to an individual's recognition of the positive outcomes of those behaviors (75). Existing research indicates contrasting effects of these perceptions: risk perception tends to decrease engagement in unhealthy behaviors (76–78), while benefit perception tends to encourage such behaviors (79–81). The role of social media is critical, as it exacerbates these perceptions by presenting both positive and negative information regarding behaviors such as vaping (82–85). This research proposes that risk and benefit perceptions play distinct roles in youth vaping behaviors and highlights the significant impact of social media on shaping these perceptions.

Additionally, perceived policy enforcement influences evaluations of risk and benefit. Previous studies in public policy (86), climate initiatives (87), and health regulations (54) have established a link between the effectiveness of policies and risk perception. Likewise, a stronger perception of policy enforcement is associated with an increased perception of benefits from compliant behaviors, such as farmland protection (8).

**Figure 1:**
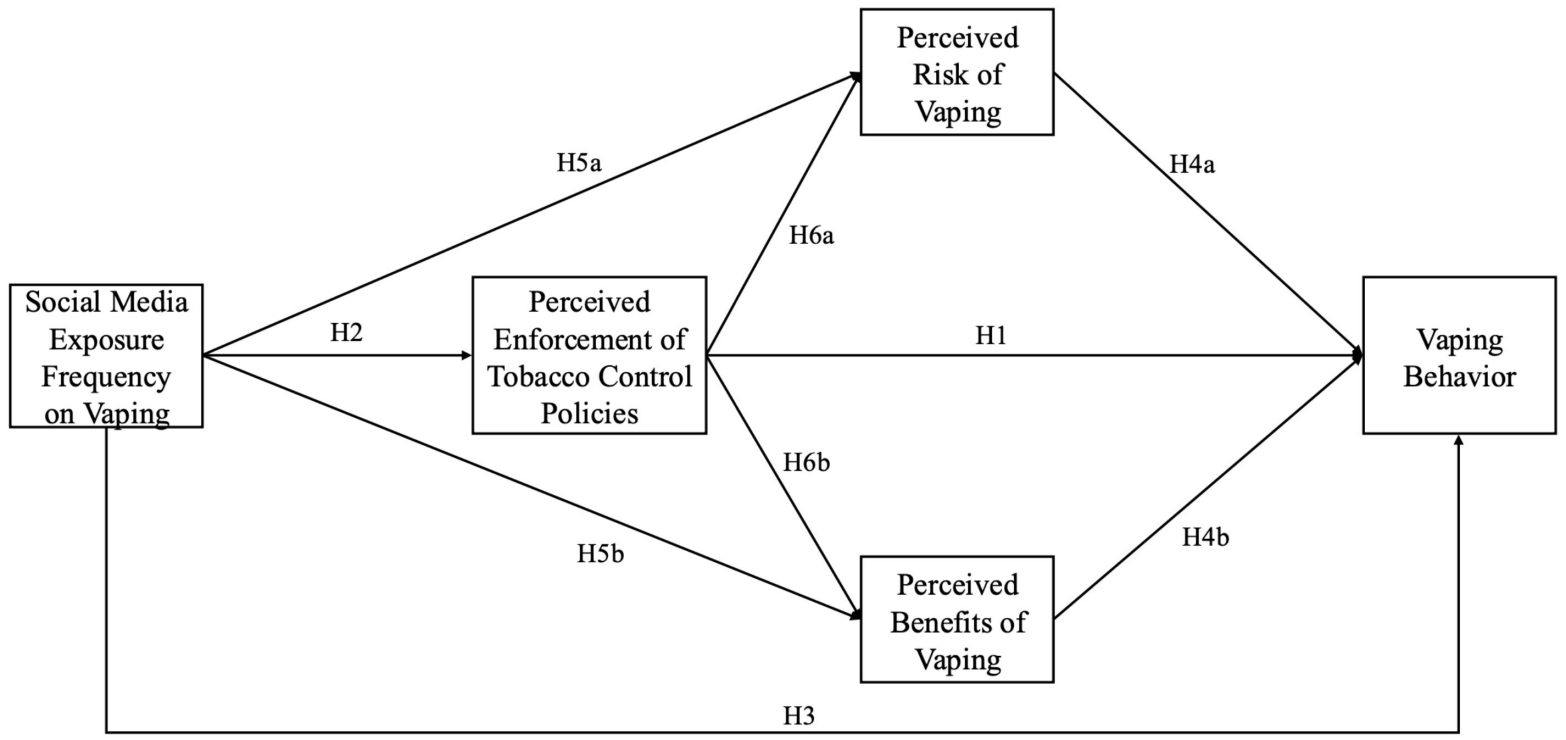
Conceptual framework.

In the context of vaping, more stringent enforcement of tobacco policies may diminish perceived benefits by indicating a higher level of harm, while simultaneously elevating risk perceptions. Within the framework of KAP model in China, this study introduces a mediation model (see Figure 1) that illustrates how social media exposure can directly and indirectly influence the likelihood of vaping among youth, with perceived policy enforcement, risk, and benefit perceptions serving as mediators. These mediators provide insight into how external factors translate into behavioral outcomes.

### 2.5 Hypothesis and research questions

Drawing upon theoretical frameworks and existing research, as well as considering the context of perceptions regarding policy implementation and the social media usage in this study, we develop our research hypotheses and questions. Initially, concerning the direct association between PPE and youth vaping, we propose:

*H1: The level of perceived enforcement of tobacco control policy exerts a negative influence on the vaping behaviors of young people*.

Secondly, we consider social media to be an essential source of information and propose a hypothesis regarding its influence on PPE and vaping behavior. In this context, we also develop two research questions that specifically examine the effects of social media on these two variables. They are:

*H2: The frequency of social media exposure to vaping positively influences young people's perceptions of tobacco control policy enforcement*.
*H3: The frequency of social media exposure related to vaping increases the likelihood of adolescent vaping behavior*.
*RQ1: Which types of Chinese social media impact young people's perceptions of tobacco control policy enforcement, and what are the characteristics of the information disseminated on these platforms?*
*RQ2: Which types of Chinese social media most significantly influence adolescent vaping behavior, and what are the characteristics of the pertinent social media messages?*

The two variables previously mentioned not only exert independent effects on vaping behavior but also contribute to the formation of individuals' beliefs concerning electronic cigarettes. In this context, we investigate two critical beliefs—perceived benefits and perceived risks—and their impact on vaping behavior, thereby establishing pertinent research hypotheses. Furthermore, this research aims to establish hypotheses that investigate the direct relationship between these two beliefs and the perception of policy enforcement. In addition, we seek to explore potential mediating effects through specific research questions. Accordingly, we present the following research hypotheses and questions:

*H4: A higher level of (a) perceived risk/(b) perceived benefits associated with vaping among youth will correlate with (a) a lower/(b) higher likelihood of engaging in vaping behavior*.
*H5: The frequency of youth's exposure to vaping-related content on social media may (a) negatively affect their perceived risk of vaping, and (b) positively affect their perceived benefits of vaping*.
*H6: A high level of perceived enforcement of tobacco control policies among youth (a) positively influences the perceived risk of vaping and (b) negatively influences the perceived benefits of vaping*.
*RQ3: Do the perceived enforcement of tobacco control policies and the perceived risks and benefits of vaping act as mediators in the relationship between social media exposure and vaping behavior?*

## 3 Methods

### 3.1 Data and sample

Based on a study investigating the prevalence of e-cigarette usage in China (68), we utilized G^*^Power software to determine the necessary sample size. This computation was performed with thorough consideration to essential parameters, such as the proportion of e-cigarette users, the acceptable margin of error, and the probability of committing a Type I error, etc. Consequently, we concluded that the sample size should not be <478 participants. The sample must consist of individuals who are at least 18 years of age, as this is the legal age at which Chinese citizens are permitted to purchase e-cigarettes. The current study involved a sample of 724 participants, recruited through random sampling from a population of young individuals aged 18–30 in Guangdong Province, China. Recruitment took place via an online survey administered by *Jishuyun Big Data*, a data service provider, during the period from July to September 2024. The recruitment process utilized various methods, including telephone calls, emails, WeChat QR codes, and website invitations, while initially gathering basic demographic information to ensure the sample's representativeness and validity. Once the representative sample was established, participants were invited to anonymously access the survey website using their mobile devices and complete the questionnaire. Before completing the questionnaire, it was necessary for all participants to carefully read and sign an informed consent form. Following the submission of their responses, participants were provided with information regarding the purpose of the research. It is important to note that, due to privacy considerations, the study did not collect data concerning individuals' mental health or other substance use. Detailed demographic information about the sample is presented in the results section.

### 3.2 Measurements

#### 3.2.1 Dependent variable

Vaping Behavior was measured by asking participants whether they smoked e-cigarettes (1 = yes, 0 = no) (88) (*M* = 0.515, *SD* = 0.500).

#### 3.2.2 Perceived enforcement of tobacco control policy

Perceived policy enforcement was measured by a single-item, in which respondents were instructed to indicate their subjective perception of policy enforcement of Tobacco Control Policy within their respective geographical areas (89). Response options ranging from 0 = not at all, 5 = moderate, 10 = very strict (*M* = 6.350, *SD* = 2.192).

#### 3.2.3 Social media exposure frequency on vaping

Social Media Exposure Frequency was measured by eleven questions adapted from previous research (90). The eleven items include: Over the past 6 months, how frequently have you consumed information or advertisements pertaining to e-cigarettes on (1) Weibo, (2) Wechat moments (posted or forwarded by other friends), (3) WeChat official account, (4) WeChat Channels, (5) REDnote, (6) Tiktok, (7) Kwai, (8) Bilibili, (9) Zhihu, (10) Baidu Tieba, (11) Social Media Outside China (e.g., Facebook, YouTube, Instagram, X)? Responses were scored on a five-point scale (1 = I never have, 2 = Monthly, 3 = Every few weeks, 4 = Weekly, 5 = Daily) (*M* = 1.503, *SD* = 0.846, Cronbach's alpha = 0.883).

#### 3.2.4 Perceived risk of vaping

Perceived risk was measured by fourteen questions, drawn from prior research (91). A 7-point Likert scale was used as the response format, ranging from 1 (“totally disagree”) to 7(“totally agree”), with 4 representing “neither agree nor disagree.” Based on the actual situation in China, we eliminated items within the scale that were incongruent with the Chinese context, subsequently retaining fourteen questions post-deletion. The fourteen items include: (1) E-cigarettes contain toxic chemicals. (2) The nicotine in liquid cartridges for e-cigarettes is toxic to small children and pets. (3) E-cigarettes heat a mixture of propylene glycol, nicotine, and flavoring. (4) E-cigarettes contain some of the same toxins as regular cigarettes, such as formaldehyde. (5) There is risk in inhaling the hot mix of chemicals (propylene glycol, glycerin, and nicotine) contained in e-cigarettes. (6) Nicotine is addictive, regardless of whether ingested through e-cigarettes or regular cigarettes. (7) Dual use of regular cigarettes and e-cigarettes places the smoker/vaper at risk for heart problems, lung problems, and cancer. (8) Many people who start vaping smoke cigarettes as well. (9) There are more effective ways to quit smoking than e-cigarettes. (10) Kids who use e-cigarettes are more likely to continue smoking. (11) Children and pets can become seriously ill if they drink or touch e-cigarette fluid. (12) Many local communities have started to ban the use of e-cigarettes wherever tobacco cigarettes are prohibited. (13) Liquid cartridges for e-cigarettes contain nicotine. (14) “Vaping” (smoking e-cigarettes) can lead to smoking more regular cigarettes (*M* = 4.724, *SD* = 0.888, Cronbach's alpha = 0.827).

#### 3.2.5 Perceived benefits of vaping

Perceived benefits was measured by nine questions derived from previous research (91). A 7-point Likert scale was used as the response format, ranging from 1 (“totally disagree”) to 7(“totally agree”), with 4 representing “neither agree nor disagree.” Based on the actual context in China, we eliminated the items within the scale that were incongruent with the Chinese context, and retained nine questions subsequent to the deletion process. The nine items include: (1) E-cigarettes are less harmful than regular cigarettes. (2) E-cigarettes are an effective way to quit smoking regular cigarettes. (3) E-cigarettes contain fewer chemicals than regular cigarettes. (4) Kids who use e-cigarettes are more likely to quit smoking. (5) E-cigarettes can be used anywhere even indoors. (6) E-cigarette users exhale only water vapor that contains no toxins. (7) Compared to second-hand smoke from regular cigarettes, there are no known risks to second-hand vapor from e-cigarettes. (8) E-cigarettes are safe. It's tobacco-not nicotine-that makes regular cigarettes dangerous. (9) E-cigarettes do not have the same adverse effect as regular cigarettes after smoking (i.e., mouth and throat irritation, nausea/headache and dry cough) (*M* = 4.192, *SD* = 1.233, Cronbach's alpha = 0.861).

#### 3.2.6 Control variables

Control variables included respondents' age (self-report), gender (1 = male, 0 = female), education (1 = Junior high school and below, 2 = Senior high school, 3 = college diploma, 4 = bachelor's degree, 5 = master's degree, 6 = doctoral degree), annual household income (ranging from 1 = ¥0 to ¥10,000, 14 = ¥200,000 or more).

### 3.3 Data analysis

SPSS29.0 was used for data analysis. First, to investigate the direct impacts of four independent variables—namely, the frequency of exposure to social media concerning electronic cigarettes, the perceived enforcement of tobacco control policies, the perceived risks of vaping, and the perceived benefits of vaping—on the dependent variable, vaping behavior, a binary logistic regression analysis was carried out. Second, to assess the mediation models, we utilized Model 81 from the SPSS PROCESS macro (92) to produce bootstrapped confidence intervals (CIs). Third, to identify the specific social media platforms or combinations thereof that affected perceptions of tobacco control policy enforcement and vaping behavior, we conducted linear regression analyses for perceived enforcement of tobacco control policies and logistic regression analyses for vaping behavior, utilizing varying frequencies of social media exposure as independent variables.

## 4 Results

Socio-demographic characteristics are summarized in Table 1. The participants in this study are primarily within the age range of 18–30 years, exhibiting a mean age of 25.45 years. The sample is comprised of 88.4% males (*N* = 640) and 11.6% females (*N* = 84), which closely corresponds to the overall male-to-female ratio of 9:1 observed in the smoking population of China (93). Notably, 51.5% of the participants (*N* = 373) reported using electronic cigarettes. Additionally, a significant majority of the sample, 83.2%, possesses either an associate degree or a bachelor's degree. Furthermore, the annual income of the participants primarily ranges from ¥40,001 to ¥90,000, encompassing 77.5% of the sample.

**Table 1:** Sample Characteristics (*N* = 724).

**Demographic characteristics**	***M* (SD) or *N* (%)**
**Age**	25.45 (1.98)
**Sex**	
Male	640 (88.4%)
Female	84 (11.6%)
**Education**	
Less than collage	112 (15.5%)
College undergraduate	602 (83.1%)
College graduate and Higher	10 (1.4%)
**Annual income**	
<¥50,000 ($7,000)	200 (27.6%)
¥50,001 to ¥100,000($14,000)	444 (61.3%)
¥100,001 to ¥150,000($21,000)	61 (8.4%)
>¥150,000	19 (2.7%)

The conversion of RMB to USD is an approximation.

To evaluate the hypothesis of the negative association between perceived tobacco control enforcement and vaping (H1), we performed a binary logistic regression analysis. The results of the Hosmer and Lemeshow Test for the regression model indicated a satisfactory fit, with χ^2^ (8) = 13.357 and *p* = 0.10. The detailed results are displayed in Table 2. The findings reveal that the perceived enforcement of the Tobacco Control Policy did not have a statistically significant impact on youth vaping behavior (*B* = −0.023, OR = 0.977, *SE* = 0.040, 95% CI: [0.904, 1.056]). Consequently, the result does not establish a negative relationship between the perceived enforcement of tobacco control measures and vaping behavior.

**Table 2:** Binary logistic regression on vaping.

**Variables**	**Model 1**			**Model 2**		
	**B**	**Exp(B)**	***SE***	**B**	**Exp(B)**	***SE***
**Block 1: demographics**						
Sex	−0.06	0.94	0.24	0.18^*^	1.20	0.26
Age	−0.01	0.99	0.03	0.01	1.01	0.03
Income	0.11^***^	1.12	0.03	0.10^**^	1.11	0.04
Education	0.21^*^	1.23	0.11	0.18	1.19	0.12
Δ *Pseudo R^2^* = 0.039	
**Block 2: independent variables**						
Social media exposure				−0.23^*^	0.80	0.10
Perceived policy effectiveness				−0.02	0.98	0.04
Perceived risk				−0.47^***^	0.63	0.10
Perceived benefits				0.34^***^	1.40	0.07
Δ *Pseudo R^2^* = 0.118						
*Total Pseudo R^2^* = 0.157						
*−2 Log likelihood* = 912.228						

* *p* < 0.05,

** *p* < 0.01,

*** *p* < 0.001.

**Figure 2:**
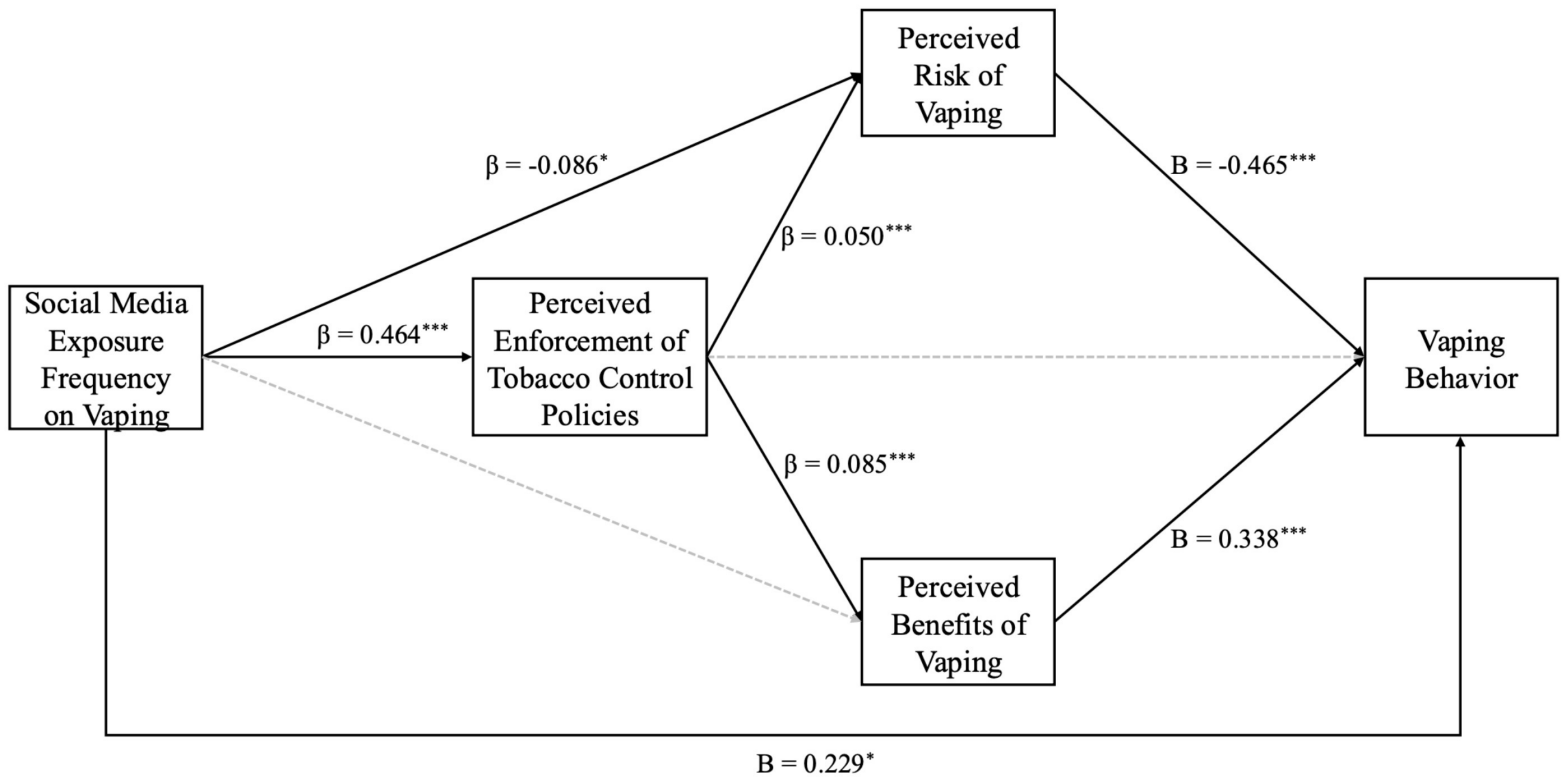
Model results. *p < 0.05, ***p < 0.001.

To investigate the positive influence of social media exposure on perceived policy enforcement (H2), we developed a mediation model (see Table 3 and Figure 2). The findings revealed that exposure to vaping content on social media had a significant impact on the perceived enforcement of tobacco control policies (β = 0.464, *SE* = 0.096, *p* < 0.001). These results suggest that increased exposure to social media content concerning e-cigarettes correlates with a heightened perception of the enforcement of tobacco control policies among young individuals. Thus, the data support the assertion that exposure to social media has a favorable impact on individuals' perceptions of policy enforcement.

**Table 3:** Results of mediation effect test.

	***b***	***SE***	**95%CI**
Social Media -> Enforcement -> Vaping	−0.011	0.020	[−0.052, 0.026]
Social Media -> Risk-> Vaping	0.040	0.025	[−0.004, 0.095]
Social Media -> Benefit->Vaping	0.006	0.020	[−0.035, 0.095]
Social Media -> Enforcement->Risk-> Vaping	−0.011	0.006	[−0.026, −0.002]
Social Media -> Enforcement->Risk-> Vaping	0.013	0.006	[0.004, 0.029]

Subsequently, we evaluate whether sustained exposure to social media content pertaining to vaping substantially enhances the probability of engaging in vaping behavior (H3). The results from both the logistic regression analysis (Table 2) and the mediation analysis (Table 3 and Figure 2) indicate that for each additional unit of exposure to social media content on vaping among adolescents, the probability of engaging in vaping behavior increased by a factor of 1.257 (*B* = 0.229, OR = 1.257, *SE* = 0.103, 95% CI: [0.904, 1.056]), indicating that social media exposure serves as a significant predictor of adolescent vaping.

We propose the hypothesis that a low perception of risks associated with vaping, in conjunction with a high perception of its benefits, may increase the likelihood of vaping behavior among adolescents (H4). Results presented in Table 2 illustrate that as the perception of risk related to vaping increases, the likelihood of engaging in vaping decreases (*B* = −0.465, OR = 0.628, *SE* = 0.095, 95% CI: [0.521, 0.757]). Conversely, an increase in the perception of benefits associated with vaping correlates with a heightened likelihood of vaping (B = 0.338, OR = 1.403, *SE* = 0.073, 95% CI: [1.216, 1.618]). Thus, empirical support has been identified indicating that risk perception negatively affects vaping behavior (H4a), while benefit perception exerts a positive influence on such behavior (H4b).

This study posits that frequent exposure to vaping-related content on social media may variably shape individuals' perceptions regarding the risks and benefits linked to vaping (H5). As depicted in Table 3 and Figure 2, increased exposure of adolescents to social media content pertaining to vaping correlates with a decreased perception of the risks involved (β = −0.086, *SE* = 0.042, *p* < 0.05). However, the data failed to prove an increase in the perception of benefits (β = 0.017, *SE* = 0.055, *p* = 0.761). As a result, there exists a negative correlation between exposure to social media and the perceived risks (H5a), while a positive relationship is identified between social media exposure and the perceived benefits of vaping (H5b).

We further investigate the impact of perceived enforcement of Tobacco Control Policy on individuals' perceptions of risk and benefit (H6). As illustrated in Table 3 and Figure 2, the findings indicated a positive relationship between both perceived risks and perceived benefits concerning the perceived enforcement of Tobacco Control Policy, thereby supporting that positive correlation between perceived enforcement and risk perception (H6a) (β = 0.050, *SE* = 0.016, *p* < 0.01). Nevertheless, the anticipated negative correlation between perceived enforcement and benefit perception (H6b) was not substantiated by the findings (β = 0.085, *SE* = 0.021, *p* < 0.001). The results indicate that the perceived enforcement of Tobacco Control Policy positively influences the perceived benefits of vaping, thereby contradicting the initial hypothesis.

The primary focus of the present study is to investigate the effectiveness of different types of social media in shaping perceptions of the enforcement of Tobacco Control Policy (RQ1) and their impact on vaping behaviors (RQ2) within the context of China. In this analysis, we designated the frequency of exposure to different forms of social media as the independent variable, while the perceived enforcement of Tobacco Control Policy served as the dependent variable for the linear regression analysis (see Model 1 in Table 4). The findings suggest that increased exposure to e-cigarette-related information on social media platforms, including (1) WeChat Moments (β = 0.114, *SE* = 0.076, *p* < 0.05), (2) REDNote (β = 0.094, *SE* = 0.079, *p* < 0.05), (3) Baidu Tieba (β = 0.169, *SE* = 0.85, *p* < 0.001), and (4) International Social Media Outside China(β = 0.083, *SE* = 0.079, *p* < 0.05), correlates with a heightened perception of Tobacco Control Policy enforcement.

**Table 4:** Regression analysis of different types of social media on policy enforcement perception and vaping prevalence.

	**Model 1 (linear regression of perceived enforcement of tobacco control policy)**				**Model 2 (binary logistic regression on vaping)**			
**Social Media**	β	***SE***	***t***	**95%CI**	**B**	***SE***	**OR**	**95%CI**
Weibo	−0.082	0.084	−1.812	[−0.317, 0.013]	−0.07	0.081	0.932	[0.796,1.092]
Wechat moments	0.114	0.076	2.516	[0.042, 0.339]	0.031	0.073	1.032	[0.895,1.189]
WeChat official account	0.001	0.084	0.026	[−0.162, 0.166]	0.038	0.08	1.039	[0.888,1.216]
WeChat Channels	0.034	0.086	0.698	[−0.109, 0.229]	−0.206	0.083	0.814	[0.691, 0.958]
REDnote	0.094	0.079	2.107	[0.011, 0.321]	0.079	0.076	1.082	[0.933,1.254]
Tiktok	0.012	0.075	0.273	[−0.127, 0.167]	−0.02	0.072	0.98	[0.852,1.128]
Kwai	−0.087	0.082	−1.775	[−0.307, 0.015]	−0.018	0.079	0.982	[0.842,1.146]
Bilibili	−0.047	0.083	−1.025	[−0.248, 0.078]	0.128	0.08	1.136	[0.972,1.328]
Zhihu	0.026	0.083	0.549	[−0.117, 0.208]	−0.008	0.08	0.992	[0.848,1.159]
Baidu Tieba	0.169	0.085	3.505	[0.132, 0.467]	0.212	0.083	1.236	[1.051,1.454]
Social Media Outside China (e.g., Facebook, Youtube, Instagram, X)	0.083	0.079	2.011	[0.004, 0.315]	0.025	0.076	1.025	[0.882,1.191]
*R^2^ = 0.08*					*−2 Log likelihood = 982.08*			
*F(11,712) = 5.536*					*pseudo R^2^ = 0.04*			

Additionally, we employed the frequency of exposure to various social media types as the independent variable and vaping behavior as the dependent variable in a binary logistic regression analysis (see Model 2 in Table 4). The results indicate two key trends: (1) a higher frequency of exposure to e-cigarette-related content on WeChat Video Channel is associated with a decreased likelihood of youth engaging in vaping behavior (*B* = −0.206, OR = 0.098, 95% CI: [0.691, 0.985]), and (2) increased exposure to e-cigarette-related content on Baidu Tieba correlates with an increased likelihood of youth participating in vaping activities (*B* = 0.212, OR = 1.236, 95% CI: [1.051, 1.454]).

The present study also examined the mediating roles of perceptions regarding policy enforcement and evaluations of the risks and benefits associated with vaping (RQ3). As illustrated in Table 3, the frequency of social media exposure to vaping significantly influences the occurrence of vaping behavior through two indirect pathways: (1) in path a, perceived enforcement of Tobacco Control Policy and perceived risk function as mediators (*b* = −0.013, *SE* = 0.006, 95% CI: [−0.028, −0.004]); and (2) in path b, perceived enforcement of Tobacco Control Policy and perceived benefits serve as mediators (*b* = 0.011, *SE* = 0.006, 95% CI: [0.002, 0.027]). Both pathways are found to be significant. This suggests that exposure to information related to e-cigarettes on social media can shape young individuals' perceptions regarding the enforcement of Tobacco Control Policy, which in turn affects their perceptions of both risks and benefits. Such perceptions may subsequently either enhance or diminish the likelihood of engaging in vaping behavior.

## 5 Discussion

### 5.1 The mediating role of perceived policy enforcement

This research examines the factors influencing vaping behavior among Chinese adolescents, focusing on the perceived enforcement of policies. Previous studies indicate that China is the world's largest consumer of tobacco products, and there is a concerning trend of decreasing age at which individuals initiate smoking, especially among the youth demographic, as highlighted by multiple sources (94–98). Also, the recent proliferation of social media has stimulated public discourse by disseminating and sharing various content, thereby altering public understanding and attitudes. This transformation, in turn, influences individuals' perceptions of policy enforcement (42–45). Therefore, a deeper understanding of how vaping behavior is influenced by social media and tobacco control policies, particularly the psychological mechanisms involved in this process, is crucial for effectively utilizing social media platforms and related policies to control smoking, especially in reducing youth vaping. The findings of this study reveal that daily exposure to vaping-related content on social media can have both direct and indirect effects on individuals' vaping behaviors. Specifically, the frequency of exposure to such content positively affects perceptions of the enforcement of Tobacco Control Policies. This perception, in turn, primes individuals' awareness of the risks and benefits associated with vaping, ultimately increasing or decreasing the likelihood of engaging in vaping behavior. Identifying this mediating pathway offers valuable insights into the psychological factors influencing youth vaping and introduces new approaches for health communication strategies aimed at intervening vaping behaviors. Additionally, framed within the KAP theory, the mediating model provides strong empirical support and theoretical contributions. The following sections will discuss the research results and their theoretical and practical implications in detail.

This study reveals that the perceived enforcement of Tobacco Control Policy does not have a significant direct effect on the likelihood of youth engaging in vaping behavior. Instead, it influences vaping through the mediation of perceived risks and perceived benefits. Specifically, the perception of effective enforcement of Tobacco Control Policy positively affects perceived risk, which in turn decreases the likelihood of vaping. Conversely, it also positively affects perceived benefits, thereby increasing the likelihood of vaping. This pathway is consistent with prior research indicating that perceptions of policy, including its effectiveness and enforcement strength, shape individuals' cognitions and attitudes, ultimately influencing their behaviors (99, 100). The interplay between perceived risk and perceived benefits, which should theoretically be opposite in terms of both causing and being caused by other variables, manifests in the pathways through which these perceptions influence vaping behavior. However, when influenced by the perceived enforcement of Tobacco Control Policy, both perceptions exhibit a positive impact. China's tobacco control policies may reflect a dual conceptualization distinguishing traditional and e-cigarettes, potentially shaping public perceptions and regulatory outcomes. When policies are perceived as strictly enforced, individuals associating regulations primarily with traditional cigarettes might view e-cigarettes as possible substitutes, which may amplify perceived benefits and possibly encourage vaping adoption. Conversely, activating policy concepts related to e-cigarettes could heighten risk perceptions, potentially discouraging their use (101, 102). This pattern may align with China's historical focus on regulating traditional cigarettes. Recent efforts to address e-cigarettes risks appear to integrate vaping governance into existing tobacco frameworks rather than establishing separate policies.

### 5.2 A potential explanation of the psychological mechanism: cognitive dissonance

From the perspective of psychological mechanisms, tobacco control policies may generate dual perceptions of e-cigarettes: awareness of health risks and belief in their substitution benefits for conventional cigarettes. This duality could create cognitive dissonance as users navigate conflicting cognitions—risk vs. benefit. When an individual's actions are at odds with both the risk perception emphasized by policies (e.g., health hazards) and the perception of e-cigarettes as beneficial (e.g., as a tool for quitting traditional cigarettes), psychological tension may arise from the inability to reconcile these conflicting beliefs. This dissonance may motivate individuals to resolve the tension through various strategies, such as altering their behavior (e.g., quitting e-cigarettes or exclusively using e-cigarettes without reverting to traditional cigarettes), selectively reinforcing one side of the cognition (e.g., emphasizing harm reduction or amplifying risks), or seeking external justification from policy authority (e.g., interpreting the policy as only requiring the cessation of traditional cigarettes, thereby rendering individual e-cigarette use permissible and rational). These strategies aim to reconcile contradictions and regain cognitive consonance. While this framework proposes explanatory psychological mechanisms, their operation along these pathways remains subject to empirical validation.

Based on the findings of this study, two practical implications emerge. First, it is crucial to strengthen the enforcement of tobacco control policies to enhance the perceived authority of these regulations concerning e-cigarettes, thereby reducing unhealthy behaviors. Second, policies should explicitly delineate the risks associated with electronic cigarettes and the relevant regulatory provisions, with the objective of maximizing public awareness regarding the potential dangers posed by e-cigarettes. It is essential to recognize that tobacco control is a comprehensive concept rather than one specifically targeting traditional cigarettes or e-cigarettes only; thus, when mention tobacco control invoke two distinct concepts—e-cigarettes and traditional cigarettes—which differentially influence risk and benefit perceptions.

### 5.3 The role of social media and its theoretical explanations

This study also examined the impact of social media exposure on vaping behavior. The results suggest that exposure to e-cigarette-related content on social media significantly enhances vaping behavior. Additionally, this exposure indirectly influences vaping through perceived enforcement of Tobacco Control Policies and the perception of risks and benefits associated with vaping. Within the framework of the KAP model, social media exposure is categorized as knowledge, which subsequently increases the likelihood of adopting specific practices. In the context of vaping, a higher frequency of exposure to e-cigarette information or advertisements on social media correlates with an increased likelihood of vaping. Typically, individuals who frequently encounter such social media content—whether through active searches, passive algorithmic feeds, or casual scanning—demonstrate heightened attention to e-cigarettes, thereby increasing their propensity to engage in vaping behavior. This direct relationship is consistent with findings from prior research (71, 103–105).

The selective exposure theory suggests that individuals shape their information environment based on their existing behaviors, which may create a reverse causal relationship that contradicts this paper's argument. For instance, young people who are inclined to use e-cigarettes might actively seek out related content on social media. This self-selection means that media exposure may result from their behavioral tendencies rather than cause them. Furthermore, this active engagement may create a reinforcing loop: individuals may strengthen their perceived rationality of the behavior through information filtering (e.g., favoring pro-e-cigarette content), social validation (e.g., interacting with like-minded users), or emotional resonance (e.g., associating e-cigarettes with being 'cool' or 'fashionable'). Thus, a loop may form where behavioral inclination leads to media exposure, which in turn reinforces that behavior. Additionally, there may be a bidirectional dynamic relationship between media exposure and behavior: initial behavioral tendencies might drive selective exposure, while the encountered information (e.g., product glorification, peer modeling) might reduce perceived behavioral costs (e.g., others' use is harmless), potentially driving the implementation or continuation of the behavior. This complex interplay requires further validation through longitudinal data or instrumental variable analysis to potentially distinguish the antecedents and consequences of media exposure.

Moreover, young adults exposed to e-cigarette-related information on social media tend to perceive a stronger enforcement of Tobacco Control Policies. This is particularly evident in the context of China, where direct e-cigarette advertisements are prohibited on social media platforms. Users often modify keywords associated with e-cigarettes to present advertising content more subtly. Such exposure to relatively discreet information fosters the perception that regulatory policies are actively governing e-cigarette use, thus enhancing their sense of enforcement. Additionally, much of the content available on social media regarding e-cigarettes comprises health-related information and discussions of tobacco control policies. Engagement with this type of content increases users' awareness of the harmful effects of e-cigarettes and their understanding of current policy measures, thereby reinforcing their perception of enforcement. Through this process, as previously noted, social media exposure shapes young adults' perceptions of risks and benefits, subsequently influencing their vaping behavior. This mediating effect elucidates a mechanism by which policy enforcement relates to social media's role in shaping vaping behavior, offering new avenues for intervention strategies targeting vaping through social media platforms.

### 5.4 The impact of information sources on different social media platforms

Our research has uncovered various direct and indirect pathways through which different types of social media information impact individuals' vaping behaviors. Specifically, exposure to e-cigarette-related content on two distinct Chinese social media platforms—WeChat Video Channels (negative impact) and Baidu Tieba (positive impact)—has been found to significantly influence the vaping behaviors of young individuals, albeit in contrasting ways. Analysis of the content on these platforms indicates that: (1) WeChat Video Channels primarily disseminate health education and policy-related information regarding the dangers of e-cigarettes, devoid of any advertising. Consequently, increased exposure to such content tends to diminish the likelihood of vaping; (2) In contrast, Baidu Tieba is characterized by a higher prevalence of advertising-oriented information about e-cigarettes, often presented in a manner that lacks health education. Users frequently employ ambiguous terminology to promote e-cigarettes in order to circumvent online censorship, which, in turn, heightens the likelihood of vaping among those frequently exposed to such content. Furthermore, the study reveals that greater exposure to e-cigarette-related information across four accessible social media platforms—WeChat Moments, REDNote, Baidu Tieba, and various international social media outside China—reinforces young people's perception of the enforcement of Tobacco Control Policies in China. Notably, platforms such as Baidu Tieba and REDNote contain a substantial number of e-cigarette advertisements, often rephrased with ambiguous keywords to evade censorship. On the one hand, exposure to these rephrased messages primes users' understanding of regulatory frameworks, heightening their perception of policy enforcement. On the other hand, the presence of e-cigarette advertisements, particularly those from international social media outside China that utilize more explicit content, may further amplify users' perception of strong policy enforcement when they encounter such information across different platforms. We contend that numerous Chinese social media platforms continue to inadequately regulate this obfuscated and homophonic content, thereby increasing the likelihood of exposure to these advertisements among young people, which subsequently influences their vaping behaviors through both direct and indirect psychological mechanisms. Therefore, the timely identification and warning of harmful health information on social media, along with the enhancement of information regulation, represent effective administrative strategies for mitigating vaping behaviors.

### 5.5 Theoretical implications

This study investigates various critical factors that influence vaping behavior, uncovering the fundamental mechanisms involved, which carry significant theoretical implications. Initially, we enhance the KAP model related to youth vaping, specifically exploring how perceptions regarding the enforcement of tobacco control policies impact individuals' views on associated risks and benefits. This enhancement broadens the pathways linked to attitudes, thereby offering new frameworks for understanding vaping behavior. Second, the study underscores the confusion that arises when applying conjoint behavioral concepts, such as tobacco control, to distinct behaviors such as e-cigarette use vs. traditional cigarette smoking. This confusion may result in different outcomes in individual decision-making processes due to the activation of various behavioral constructs. In terms of agenda-setting in health policy communication, the precise definition of concepts and the priming mechanisms are vital for influencing individuals' cognition, attitudes, and behaviors at a micro level.

### 5.6 Practical implications

This study presents practical implications in two significant dimensions. First, tobacco control policies must not only raise public awareness through a well-rounded conceptual framework but also involve targeted advocacy efforts tailored to specific behaviors and contextual factors. In the case of China, the existing tobacco control policies demonstrate extensive reach and comprehensive regulation; however, there is a pressing need to enhance enforcement mechanisms. This enhancement is essential for effectively shaping public perceptions regarding the risks associated with e-cigarette use and for mitigating vaping behaviors. Second, given that social media platforms serve as vital sources of information for young individuals, it is crucial to manage health-related information with precision. This includes minimizing ambiguity and coded messaging and improving the scope and depth of health science information dissemination. Such strategies are intended to enhance public risk perception and rectify the widespread misconception that e-cigarettes pose no harm to human health.

## 6 Conclusion and limitations

This study examines the underlying mechanisms that influence vaping behavior, focusing on factors such as exposure to social media content, the perceived enforcement of Tobacco Control Policy, perceived risks, and perceived benefits. The results demonstrate that exposure to e-cigarette-related information and advertisements on social media enhances the perceived enforcement of Tobacco Control Policy. This enhancement subsequently affects both the perceived risks and perceived benefits, thereby shaping the vaping behavior among youth. The mediating mechanisms identified in this research contribute to the expansion of the KAP theory. Furthermore, the study investigates the role of Chinese social media platforms in relation to youth vaping behavior and the perceived enforcement of Tobacco Control Policy. It finds that platforms featuring more ambiguous and coded information regarding e-cigarettes exert a more pronounced influence on youth vaping behavior compared to those with clearer content. Consequently, the regulation of information on social media platforms and the reinforcement of policy enforcement emerge as vital strategies for mitigating vaping behavior in China.

It is essential to acknowledge the limitations inherent in this study. First, the investigation into exposure to e-cigarette-related information on social media did not sufficiently differentiate among various types of content, such as specific inquiries about exposure to advertisements, health knowledge, or introductions to regulatory policies. Such distinctions would greatly enhance the understanding of the priming effects of different concepts within a precise analytical framework. Subsequent research should aim to address this differentiation. Second, this research investigates the vaping behaviors among the younger generation in China. However, due to the considerable regional differences in both economic conditions and cultural practices across this vast nation, it is essential to integrate insights from the fields of economic geography and cultural geography to provide a more nuanced understanding of the research subjects. Third, while this study utilized a cross-sectional design, it is important to note that the fluctuating rates of e-cigarette use and varying levels of public awareness regarding the hazards associated with e-cigarettes render cross-sectional surveys inadequate for establishing causal relationships. Consequently, future studies could benefit from the application of longitudinal data to elucidate the causal pathways connecting social media exposure, perceived policy enforcement, and vaping behaviors. Fourth, the study's sample was predominantly male, which, although reflecting the statistical characteristics of smoking populations in China, limits the generalizability of the findings to female users. The vaping behaviors of women, their perceptions of tobacco control policies, and their attitudes toward secondhand and thirdhand smoke may differ from those of men. A more gender-balanced sample could potentially produce different outcomes. Therefore, future research should investigate the gender disparities that influence vaping behaviors in greater depth. Last, this research did not consider mental health functioning and additional substance use as covariates, presenting a limitation. Future studies should investigate the interplay of these factors in shaping youth vaping behaviors.

## Data Availability

The raw data supporting the conclusions of this article will be made available by the authors, without undue reservation.
